# Development of an angiogenesis-promoting microvesicle-alginate-polycaprolactone composite graft for bone tissue engineering applications

**DOI:** 10.7717/peerj.2040

**Published:** 2016-05-19

**Authors:** Hui Xie, Zhenxing Wang, Liming Zhang, Qian Lei, Aiqi Zhao, Hongxiang Wang, Qiubai Li, Zhichao Chen, WenJie Zhang

**Affiliations:** 1 Institute of Hematology, Union Hospital, Tongji Medical College, Huazhong University of Science and Technology, Wuhan, China; 2 Department of Plastic and Reconstructive Surgery, Shanghai 9th People’s Hospital, Shanghai Jiao Tong University, Shanghai, China; 3 Department of Hematology, the Central Hospital of Jingzhou, Jingzhou Hubei, China; 4 Department of Hematology, the Central Hospital of Wuhan, Wuhan, China

**Keywords:** Bone tissue engineering, Angiogenesis, Mesenchymal stem cell, Microvesicle, Alginate, Polycaprolactone

## Abstract

One of the major challenges of bone tissue engineering applications is to construct a fully vascularized implant that can adapt to hypoxic environments in vivo. The incorporation of proangiogenic factors into scaffolds is a widely accepted method of achieving this goal. Recently, the proangiogenic potential of mesenchymal stem cell-derived microvesicles (MSC-MVs) has been confirmed in several studies. In the present study, we incorporated MSC-MVs into alginate-polycaprolactone (PCL) constructs that had previously been developed for bone tissue engineering applications, with the aim of promoting angiogenesis and bone regeneration. MSC-MVs were first isolated from the supernatant of rat bone marrow-derived MSCs and characterized by scanning electron microscopic, confocal microscopic, and flow cytometric analyses. The proangiogenic potential of MSC-MVs was demonstrated by the stimulation of tube formation of human umbilical vein endothelial cells *in vitro*. MSC-MVs and osteodifferentiated MSCs were then encapsulated with alginate and seeded onto porous three-dimensional printed PCL scaffolds. When combined with osteodifferentiated MSCs, the MV-alginate-PCL constructs enhanced vessel formation and tissue-engineered bone regeneration in a nude mouse subcutaneous bone formation model, as demonstrated by micro-computed tomographic, histological, and immunohistochemical analyses. This MV-alginate-PCL construct may offer a novel, proangiogenic, and cost-effective option for bone tissue engineering.

## Introduction

In bone tissue engineering, seed cells play crucial roles in secreting growth factors and directly differentiating into the target tissue (Wang et al., 2015). However, for large bone defects that typically lack initial vascularization, implanted seed cells are often located a few hundred microns away from the nearest capillary supply and thus suffer from hypoxia and undergo apoptosis, resulting in a necrotic core of the implant (Moon & West, 2008). Therefore, it is necessary to improve the proangiogenic ability of bone tissue engineering scaffolds.

Recently, three-dimensional (3D) printed scaffolds have been widely studied for tissue engineering applications owing to their precise shape design and abundant choice of components (Oryan et al., 2014). Polycaprolactone (PCL) is a biodegradable polymer which has high mechanical strength and a low rate of degradation (Shor et al., 2009). It is generally considered that the degradation, mechanical strength and biocompatibility characteristics of PCL are suitable for bone tissue engineering applications (Cheung et al., 2007; Petrie Aronin et al., 2008). However, PCL has a low cellular activity because it does not possess any biological molecules (Rath et al., 2012). To address these drawbacks, alginate is often used in combination with PCL scaffolds because it is structurally similar to extracellular matrix and can encapsulate various bioactive molecules (Rufaihah & Seliktar, 2015). Growth factors, such as vascular endothelial growth factor (VEGF) and bone morphogenetic proteins (BMPs), can easily be incorporated into hydrogels and then seeded onto porous 3D printed scaffolds, thereby promoting angiogenesis and cell differentiation. For example, Kim, Jung & Kim (2013) investigated the osteoinductive potential of PCL scaffolds, and showed that it can be enhanced by coating PCL with alginate and BMP-2. However, the potential of growth factors in bone tissue engineering applications has been limited by their short half-life, low protein stability, high cost of production and restricted spatialtemporal effects due to lack of appropriate delivery approaches (Mitchell et al., 2016).

Microvesicles (MVs) are spheroidal particles enclosed by a phospholipid bilayer with a diameter typically ranging from 30 to 1,000 nm (Ratajczak et al., 2006; Lee et al., 2011). Although MVs comprise a heterogeneous group, there are two common types: exosomes and microparticles. Exosomes originate from the endosomal compartment by fusion of multivesicular bodies with the plasma membrane, while microparticles (also called ectosomes) form by direct budding from the plasma membrane (Cocucci, Racchetti & Meldolesi, 2009; Gatti et al., 2011). During their developmental process, MVs ‘hijack’ both the membrane components (including antigens, receptors, and lipid rafts) and the cytoplasmic contents (including proteins, lipids, and nucleic acids) of their parent cells (Ratajczak et al., 2006). Upon release from the parent cells, MVs may interact with or enter their target cells and deliver their bioactive cargoes to them (Van der Pol et al., 2012). Cells may be changed by direct interactions, transfer of cell surface receptors, or epigenetic reprogramming (Thery, Ostrowski & Segura, 2009; Quesenberry et al., 2014).

Accumulating evidence indicates that mesenchymal stem cell-derived MVs (MSC-MVs) possess potent proangiogenic potential (Zhang et al., 2012; Bian et al., 2014; Chen et al., 2014; Lopatina et al., 2014). It has been reported that MSC-MVs can promote the proliferation, migration, and tube formation ability of endothelial cells in vitro (Zhang et al., 2012; Bian et al., 2014; Lopatina et al., 2014). In addition, the proangiogenic potential of MSC-MVs was demonstrated in several ischemic models in vivo (Zhang et al., 2012; Bian et al., 2014). Moreover, analyses of MSC-MVs revealed the enrichment of angiogenesis-promoting growth factors and angiogenesis-associated mRNAs and miRNAs (Chen et al., 2014; Eirin et al., 2014). As MVs are generally considered to be miniature versions of their parent cells, and because MSCs incorporated into hydrogels have been successfully used in regenerative medicine (Yao et al., 2015), we hypothesized that MSC-MVs could also be incorporated into hydrogels to promote neovascularization.

**Figure 1:**
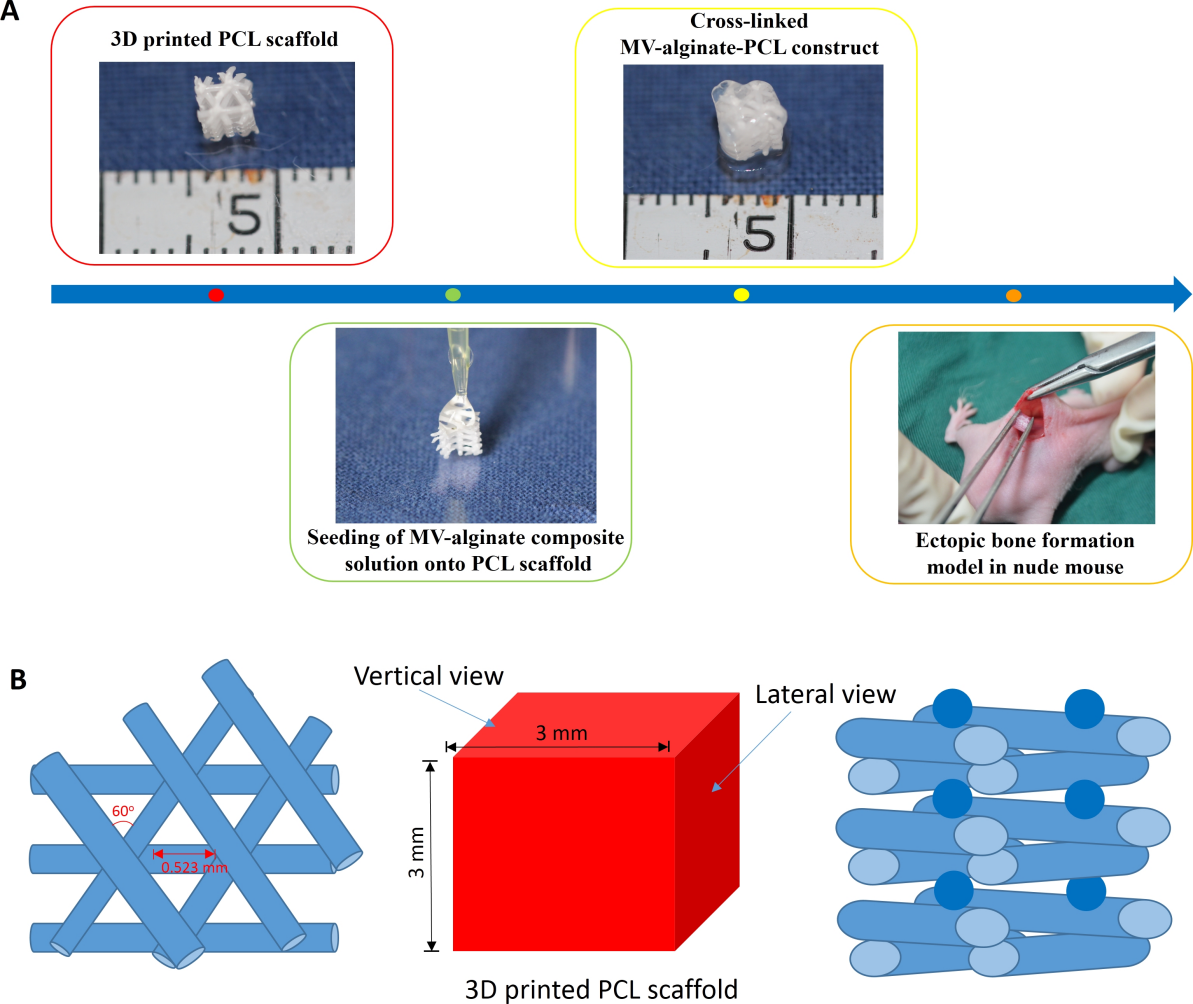
Schematic design of the fabrication of MV-alginate-PCL constructs. (A) The procedures for fabricating the MV-alginate-PCL construct. MSC-MVs were isolated and resuspended with sodium alginate solution. Sterilized PCL scaffolds were loaded with MV-alginate composite solution and cross-linked with CaCl_2_ solution. The MV-alginate-PCL constructs were implanted subcutaneously into nude mice for micro-CT, histological and immunohistochemical analyses. (B) A sketch of the structure of the 3D printed PCL scaffold.

In the present study, we developed an MV-alginate-PCL construct with enhanced proangiogenic ability for bone tissue engineering applications. The characteristics and proangiogenic properties of MVs released from rat bone marrow-derived MSCs (BMSCs) were examined *in vitro*. The procedures for fabricating the MV-alginate-PCL construct are shown in Fig. 1A. Subsequently, the effects of the MV-alginate-PCL construct on vascularization and tissue-engineered bone regeneration in vivo were investigated in a subcutaneous bone formation model in nude mice.

## Materials and Methods

### Generation of 3D printed porous PCL scaffolds

A PCL scaffold with a honeycomb-like pattern was fabricated using a fused deposition modeling technique, leading to triangular pores with a porosity of 70% and an average pore size of 0.523 mm, as previously described (Zhang et al., 2010). The PCL scaffold was cut into 3-mm cubes and immersed in 75% ethanol for 2 h. Then the scaffolds were washed three times with PBS and dried at room temperature. A sketch of the structure of PCL scaffold is shown in Fig. 1B.

### Cell culture

#### Primary culture of BMSCs

All animal experiments were approved by the Ethical Committee of Tongji Medical College, Huazhong University of Science and Technology. Bone marrow was harvested from male Sprague–Dawley rats aged 2–3 weeks. Bone marrow was flushed out from the femurs and tibias with Dulbecco’s modified Eagle’s medium (DMEM; Hyclone, Logan, UT, USA) containing 10% fetal bovine serum (FBS; Hyclone) using a 1-mL syringe. The cells were centrifuged at 500 × g for 5 min. The cell pellet was resuspended in 10 mL of DMEM supplemented with 10% FBS (Hyclone) and 1% penicillin-streptomycin antibiotic (Gibco, Carlsbad, CA, USA), and the cells were seeded in a culture dish. After 48 h, the medium was changed and nonadherent cells were discarded. Cell passaging was performed until the monolayer of adherent cells reached 70–80% confluence. All of the experiments described below were performed using BMSCs from the third to fourth passage.

#### Culture of human umbilical vein endothelial cells

Human umbilical vein endothelial cells (HUVECs) were purchased from the American Type Culture Collection (ATCC, Rockville, MD, USA). The cells were cultured in DMEM supplemented with 10% FBS and 1% penicillin–streptomycin antibiotic in a humidified incubator under an atmosphere of 5% CO_2_/95% air at 37 °C. Cell passaging was performed when the monolayer of adherent cells reached 90% confluence.

### Characterization of BMSCs

#### Trilineage differentiation of BMSCs

The trilineage differentiation potentials of BMSCs were measured as previously described (Zhang et al., 2009). All chemicals were purchased from Sigma (St. Louis, MO, USA) unless otherwise stated. For osteogenic induction, BMSCs were cultured in osteogenic differentiation medium (DMEM supplemented with 10 mM *β*-glycerophosphate, 0.1 µM dexamethasone, and 50 µM ascorbic acid) for up to two weeks, with the medium changed twice a week. The extracellular accumulation of calcium was assayed by alizarin red staining. For adipogenic induction, BMSCs were cultured in adipogenic differentiation medium (DMEM supplemented with 5 µg/mL insulin, 200 µM indomethacin, 1 µM dexamethasone, and 0.5 mM 3-isobutyl-1-methylxanthine) for 3 weeks, with the medium changed twice a week. The presence of lipid vacuoles was confirmed by oil red O staining. For chondrogenic induction, 1 × 10^6^ BMSCs were pelleted by centrifugation at 500 × *g* for 5 min in a 15 mL centrifuge tube and incubated overnight in a humidified incubator with 5% CO_2_ at 37 °C. The pelleted BMSCs were then cultured in DMEM supplemented with 0.1 µM dexamethasone, 0.17 mM ascorbic acid, 1.0 mM sodium pyruvate, 0.35 mM L-proline, 1% insulin-transferrin sodium-selenite, 1.25 mg/mL bovine serum albumin, 5.33 µg/mL linoleic acid, and 0.01 µg/mL transforming growth factor-*β* (Cell Science, Canton, MA, USA) for four weeks, with the medium changed twice a week. The micromass pellets were formalin-fixed, paraffin-embedded, and cut into 10-µm sections. The sections were dewaxed and rehydrated before safranin O staining.

#### Immunophenotype

BMSCs were fixed in 10% formalin for 15 min and washed with phosphate-buffered saline (PBS). The expression of CD73, CD105, CD29, CD44, CD34, and CD45 was detected using rabbit anti-rat CD73, CD105, CD29, CD44, CD34, and CD45 monoclonal antibodies (Abcam, Cambridge, UK), respectively, followed by goat anti-rabbit IgG conjugated with fluorescein isothiocyanate (FITC; Invitrogen, Carlsbad, CA, USA). 4′6-Diamidino-2-phenylindole (DAPI; Beyotime, Beijing, China) was used for staining nuclei.

### Isolation and characterization of MSC-MVs

MSC-MVs were harvested from the supernatant of BMSCs after 24 h of culture in DMEM without FBS, as described previously (Hergenreider et al., 2012) with some modifications. After centrifugation at 2, 000 × *g* for 20 min to remove cellular debris, the supernatant was centrifuged at 20, 000 × *g* for 1 h at 4 °C. The supernatant was then discarded, and the pelleted MVs were washed with ice-cold PBS and pelleted again by centrifugation at 20, 000 × *g* for 1 h at 4 °C. Finally, the supernatant was discarded, and the pelleted MVs were resuspended with PBS and stored at −80 °C until further experiments.

The morphology of MSC-MVs was visualized using a scanning electron microscope (SEM; Hitachi, Tokyo, Japan), as previously described (Sokolova et al., 2011) with some modifications. Briefly, MSC-MVs were fixed with 2.5% glutaraldehyde in PBS. After 2 h of fixation, glutaraldehyde was discarded, and the fixed MVs were washed twice with PBS and pelleted by centrifugation at 20, 000 × *g* for 1 h at 4 °C. MVs were then dehydrated in a series of ethanol solutions with increasing concentrations. The samples were dried at room temperature and then subjected to gold-palladium sputtering, followed by SEM analysis. For confocal microscopic analysis, MVs were stained with carboxyfluorescein succinimidyl ester (CFSE; Beyotime) in accordance with the manufacturer’s instructions, and then observed with a confocal microscope (Leica, Wetzlar, Germany).

The phenotypic profile of MSC-MVs was determined by flow cytometry with an array of antibodies commonly used for MSC identification, as previously described (Sun et al., 2014) with some modifications. Standard microbeads with a diameter of 1 µm (Sigma) were used to set the upper size limit for MVs. Calcein AM (Molecular Probes, Eugene, OR, USA) was used to avoid the staining of cell debris. MVs were co-stained with calcein AM and phycoerythrin (PE) or peridinin-chlorophyll-protein (PerCP)-conjugated anti-CD73, -CD105, -CD29, -CD44, -CD90, -CD34, and -CD45 antibodies (BD Biosciences, San Jose, CA, USA), and analyzed using a FACSAria II flow cytometer (BD Biosciences). MVs were defined by the presence of calcein AM positivity and forward scatter signals lower than those of the 1-µm standard microbeads. Flow cytometric data were analyzed using FLOW JO software version 7.6 (Tree Star Inc., Ashland, OR, USA).

### Tube formation assay

HUVECs (5 × 10^4^ cells/well) were seeded onto the Matrigel (BD Biosciences)-coated wells of a 24-well plate and cultured in serum-free DMEM in the presence of various concentrations of MVs (1, 20, and 50 µg/mL) or PBS (control). Three replicated wells were set up for each group. Tube formation was examined using a phase-contrast microscope (Olympus, Tokyo, Japan) and the total length of the network was evaluated in five randomly selected fields for each well. The total length of the network was measured using Image-Pro Plus 6.0 software (Media Cybernetics, Silver Spring, MD, USA), and expressed as a ratio to that of the respective control.

### Bone regeneration *in vivo*

To investigate the effects of the MV-alginate-PCL constructs on promoting vascularization and tissue-engineered bone regeneration, four groups were prepared and implantation was performed subcutaneously into 4-week-old male nude mice (*n* = 10 per group). The four groups were as follows: BMSC-MV-alginate-PCL group; BMSC-alginate-PCL group; MV-alginate-PCL group; and Alginate-PCL group. For the BMSC-MV-alginate-PCL group, BMSCs were subjected to osteogenic induction (as described above) for 2 weeks in culture dishes. Thawing of MSC-MVs was carried out in a preheated water bath at 37 °C and the thawed MVs were pelleted by centrifugation at 20, 000 × *g* for 1 h at 4 °C. The pelleted MVs were resuspended with 1.5% sodium alginate (Sigma, product number W201502) solution at a final MV density of 1 µg/µL. Osteodifferentiated BMSCs were then harvested and mixed with the MV-alginate composite solution at a final density of 2 × 10^7^ cells/mL. Twenty microliters of the BMSC-MV-alginate composite solution was seeded onto each PCL scaffold, and the constructs were completely immersed in 100 mM CaCl_2_ solution for about 2 min to allow cross-linking. To investigate the distribution of BMSCs in BMSC-MV-alginate-PCL constructs, BMSCs were labeled with 1,1′-dioctadecyl-3,3,3′,3′-tetramethylindocarbocyanine dye (CM-Dil; Invitrogen, Carlsbad, CA, USA) following the manufacturer’s instructions, and then mixed with MV-alginate composite solution and seeded onto PCL scaffold. The constructs were immersed in 100 mM CaCl_2_ solution for 2 min and then observed by phase-contrast microscopy and confocal microscopy to determine whether the seeded cells were homogeneously distributed throughout the scaffold. For the BMSC-alginate-PCL group, BMSCs were subjected to osteogenic induction for 2 weeks, harvested, and mixed with 1.5% sodium alginate solution at a final density of 2 × 10^7^ cells/mL. Twenty microliters of the cell-alginate composite solution was then seeded onto each PCL scaffold and the constructs were completely immersed in 100 mM CaCl_2_ solution for about 2 min to allow cross-linking. For the MV-alginate-PCL group, pelleted MVs were resuspended with 1.5% sodium alginate solution at a final MV density of 1 µg/µL. Each PCL scaffold was then loaded with 20 µL of the MV-alginate composite solution and cross-linked with CaCl_2_ solution. For the Alginate-PCL group, each PCL scaffold was loaded with 20 µL of 1.5% sodium alginate solution and cross-linked with CaCl_2_ solution. After 1 and 2 months of implantation, the animals were euthanized by an overdose of anesthesia and specimens were harvested for micro-computed tomographic (micro-CT), histological, and immunohistochemical analyses.

### Micro-CT analysis

Micro-CT analysis was performed with a µCT-80 machine (Scanco Medical, Bassersdorf, Switzerland). Samples were fixed in 4% formalin and placed in the sample holder. The region of interest was set as a cylinder (36 mm in diameter and 5 mm in height) including all of the samples from a single group. The samples were three-dimensionally reconstructed, and the parameters of bone volume (BV) and bone volume per tissue volume (BV/TV) were obtained with micro-CT auxiliary software (Volume Graphics GmbH, Heidelberg, Germany).

### Histological and immunohistochemical analyses

After the micro-CT analysis, the specimens were decalcified in 10% ethylene diamine tetraacetic acid solution for one week, dehydrated through an ethanol series, and embedded in paraffin for sectioning. The specimens were cut into 10-µm sections, mounted on glass slides, and stained with hematoxylin and eosin (HE).

Immunohistochemical staining was performed on 10-µm sections. Antigen retrieval was performed prior to incubation with a rabbit anti-mouse CD31 (commonly used endothelial marker) monoclonal antibody (Abcam). The sections were then incubated with a horseradish-peroxidase-conjugated goat anti-rabbit antibody (Invitrogen), followed by color development with diaminobenzidine tetrahydrochloride (Santa Cruz Biotechnology, Santa Cruz, CA, USA) as the substrate. Five randomly selected 200 × fields in each slice (n = 3/group) were captured using a light microscope (Olympus). The number of CD31-positive vessels was calculated using Image-Pro Plus 6.0 software (Media Cybernetics).

### Statistical analysis

All data are presented as means ± standard deviation and were analyzed by one-way analysis of variance. Values of *p* < 0.05 were considered statistically significant.

## Results

### Characterization of BMSCs

**Figure 2:**
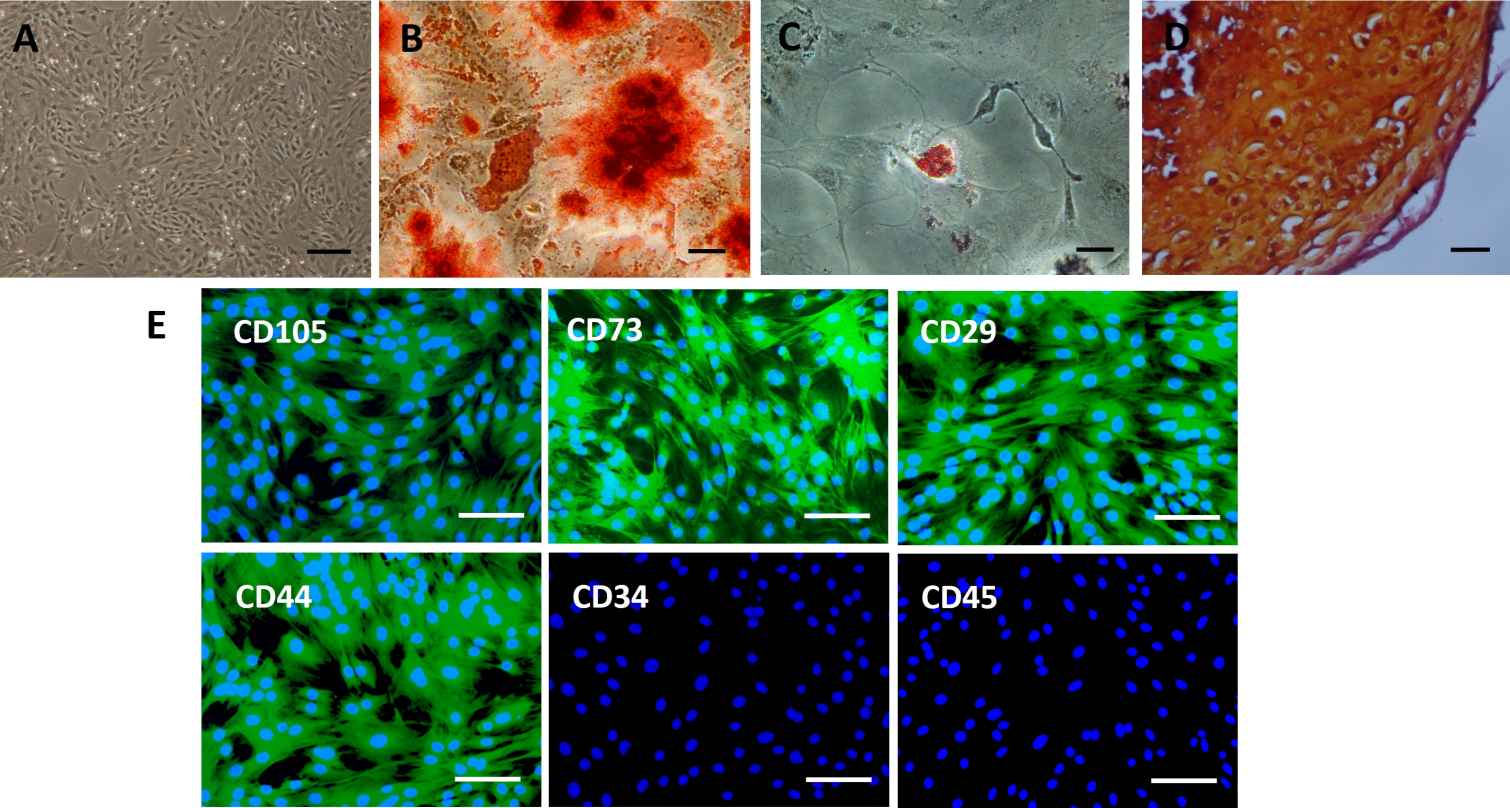
Characterization of rat BMSCs. (A) Basic morphology of rat BMSCs. Scale bar: 250 µm. (B) BMSCs underwent osteogenic differentiation (demonstrated by alizarin red staining). Scale bar: 50 µm. (C) BMSCs underwent adipogenic differentiation (demonstrated by oil red O staining). Scale bar: 50 µm. (D) BMSCs underwent chondrogenic differentiation (demonstrated by safranin O staining). Scale bar: 25 µm. (E) Immunofluorescent staining of rat BMSCs showed that they were positive for CD73, CD105, CD29 and CD44 and negative for CD34 and CD45. Scale bars: 100 µm.

Culture-expanded rat BMSCs displayed a spindle-like morphology (Fig. 2A). Extracellular calcium deposition was confirmed by alizarin red staining after 2 weeks of osteogenic induction of BMSCs (Fig. 2B). Exposure to adipogenic induction medium resulted in the accumulation of lipid vacuoles in the cytoplasm of BMSCs, as identified by oil red O staining (Fig. 2C). After four weeks of cell pellet culture in chondrogenic induction medium, BMSCs underwent chondrogenic differentiation, as confirmed by safranin O staining (Fig. 2D). Immunofluorescence staining revealed an immunophenotype that was positive for mesenchymal markers (CD73 and CD105) and cell adhesion molecules (CD29 and CD44) and negative for hematopoietic markers (CD34 and CD45) (Fig. 2E).

### Characterization of MSC-MVs

**Figure 3:**
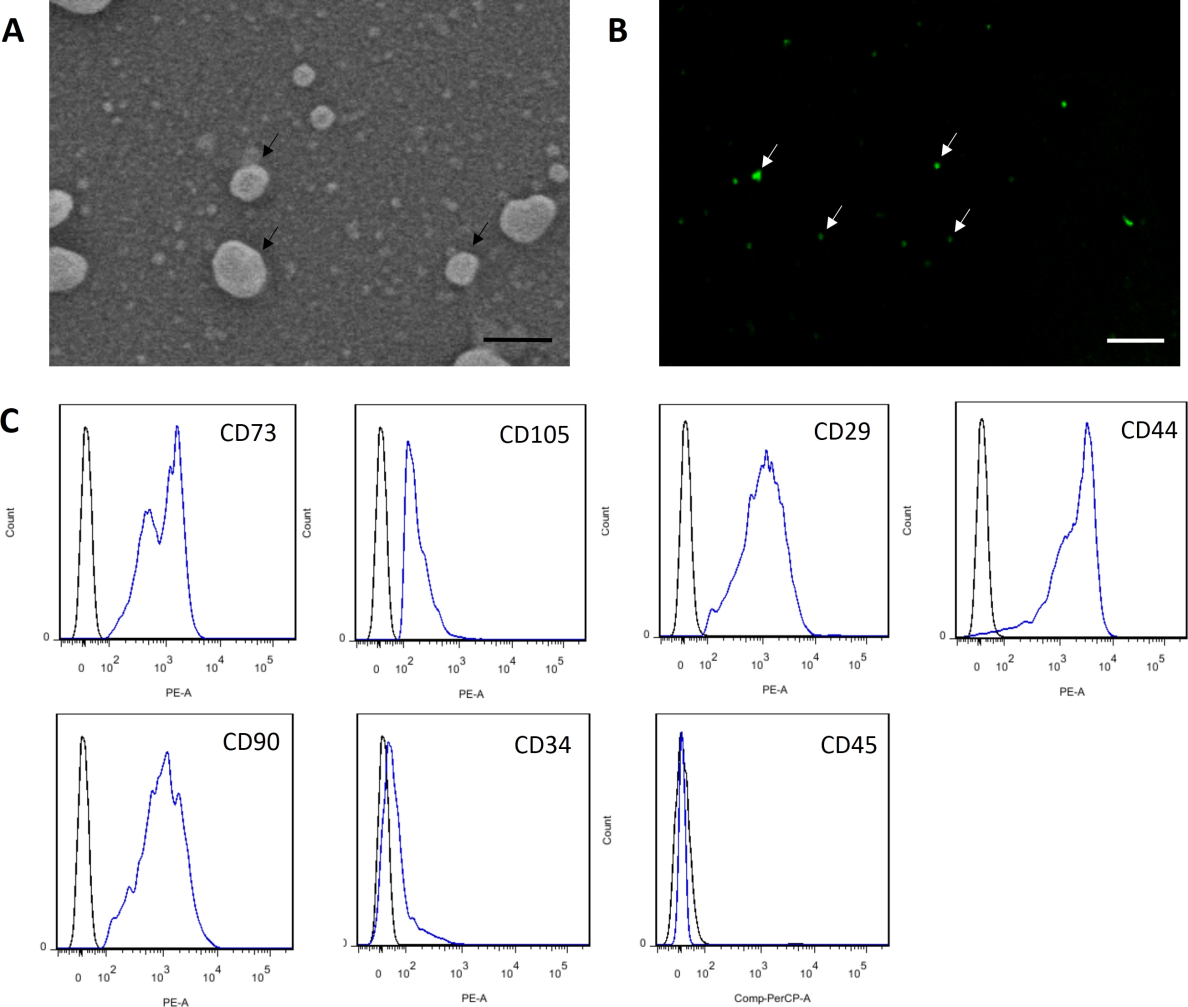
Characterization of MSC-MVs. (A) An SEM image revealing MSC-MVs (arrows) as spheroidal vesicles 100–1,000 nm in diameter. Scale bar: 500 nm. (B) Confocal microscopy image of CFSE-stained MSC-MVs (arrows) with green fluorescence. Scale bar: 7.5 µm. (C) Flow cytometric analysis of MSC-MVs revealed that they were positive for CD73, CD105, CD29, CD44 and CD90 and negative for CD34 and CD45.

MSC-MVs showed a spheroidal shape with a diameter of 100–1,000 nm when observed under an SEM (Fig. 3A). They could be observed by confocal microscopy after staining with the fluorescent dye CFSE (Fig. 3B). For flow cytometric analyses, 1-µm standard microbeads were used as an internal size standard and calcein AM was used to avoid concomitant staining of cellular debris. Only particles with forward scatter signals below the level of the 1-µm standard microbeads and positively stained for calcein AM were defined as intact MVs. Our data showed that MSC-MVs exhibited an immunophenotype that was positive for CD73, CD105, CD29, CD44, and CD90 and negative for CD34 and CD45 (Fig. 3C).

### MSC-MVs promote tube formation of HUVECs *in vitro*

**Figure 4:**
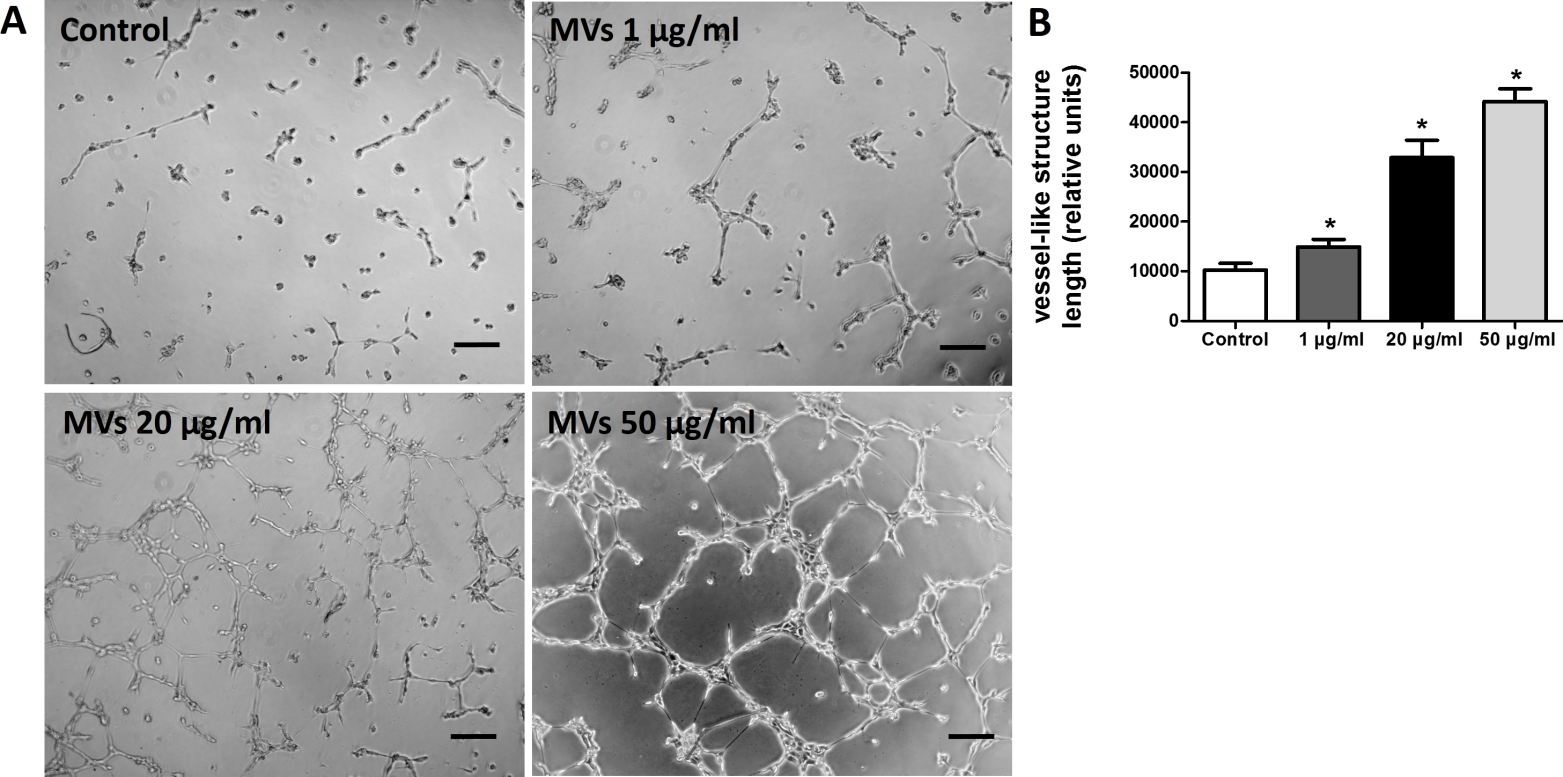
MSC-MVs promoted tube formation of HUVECs. (A) Representative images of tube formation assay in Matrigel. Scale bars: 200 µm. (B) Quantitative analysis of total length of vessel-like structures. Three replicated wells were set up for each group and five randomly selected views from each well were analyzed. *p < 0.05 vs. control.

The effect of MSC-MVs on *in vitro* capillary network formation was determined by a tube formation assay in Matrigel. Microscopic observation revealed that the difference between HUVECs treated with or without MSC-MVs was evident after 12 h of incubation. Therefore, the total length of the network structure was analyzed at the time point of 12 h. The results showed that MSC-MVs stimulated tube formation of HUVECs in a dose-dependent manner (Fig. 4), suggesting that MVs secreted by rat BMSCs could promote angiogenesis *in vitro*.

**Figure 5:**
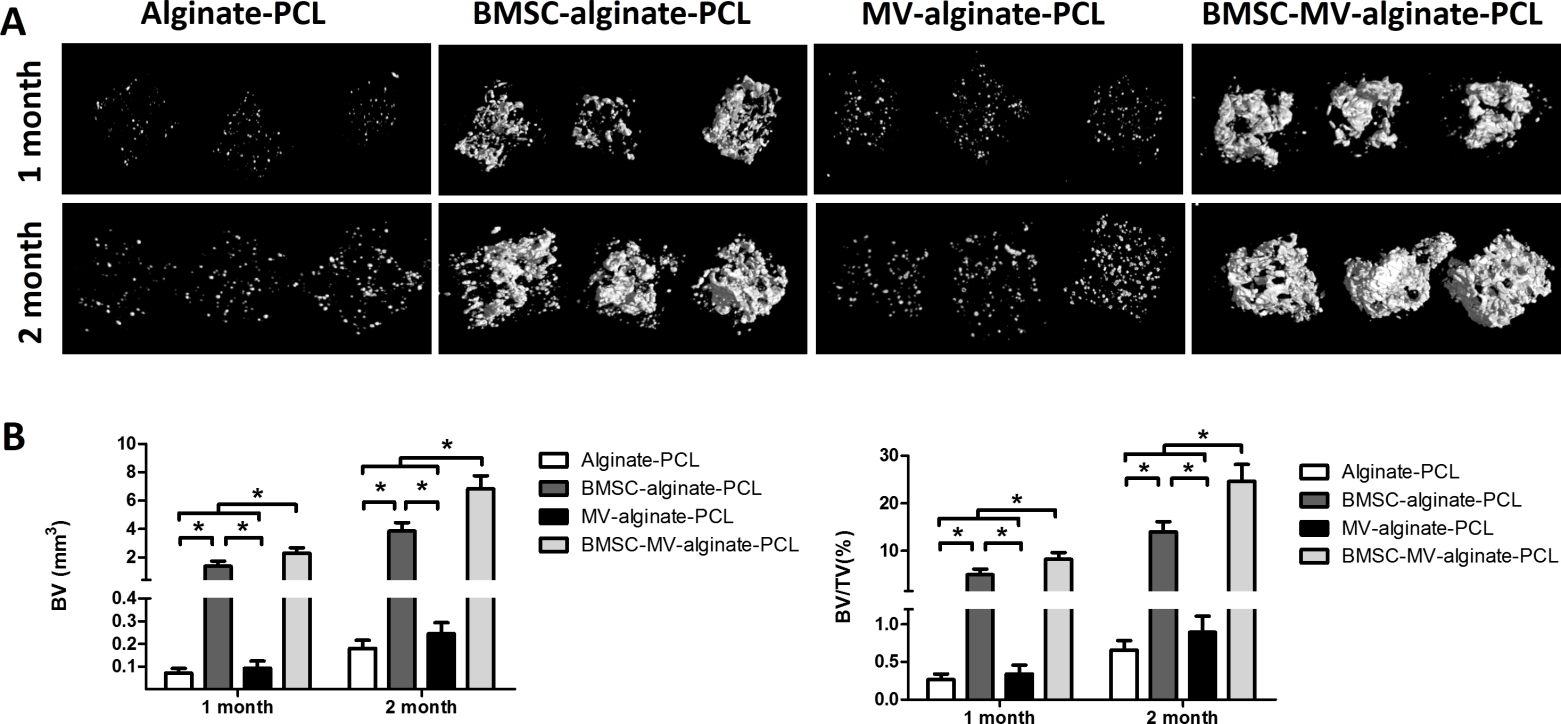
Micro-CT analysis of bone formation. (A) 3D reconstruction of micro-CT images of the specimens from all of the four groups. (B) Quantitative analysis of bone volume (BV) and bone volume/tissue volume (BV/TV) in each group at 1 and 2 months (*n* = 5/time point). *p < 0.05.

### MV-alginate-PCL constructs promote tissue-engineered bone regeneration *in vivo*

To investigate the distribution of the seeded cells in BMSC-MV-alginate-PCL construct, BMSCs were labeled with CM-Dil and the whole construct was observed by phase-contrast microscopy and confocal microscopy. As shown in Figs. S1A and S1B, BMSCs were incorporated in alginate and the alginate solution filled up the triangular pores of PCL scaffold. Additionally, confocal microscopy showed that CM-Dil labeled BMSCs were homogenously distributed around the PCL struts (Figs. S1C and S1D). To determine whether MV-alginate-PCL constructs could promote vascularization and tissue-engineered bone regeneration, four groups were prepared and implantation was performed subcutaneously into nude mice. After one and two months of implantation, samples from all groups were harvested and scanned by micro-CT to evaluate bone formation. As shown in Fig. 5, after one month of implantation, the BV and BV/TV in the BMSC-MV-alginate-PCL group (2.29 ± 0.38 mm^3^, 8.18% ± 1.42%) were significantly increased compared with those in the BMSC-alginate-PCL (1.37 ± 0.36 mm^3^, 4.95% ± 1.12%), MV-alginate-PCL (0.09 ± 0.03 mm^3^, 0.34% ± 0.12%), and Alginate-PCL (0.07 ± 0.02 mm^3^, 0.27% ± 0.08%) groups. The differences between the BMSC-MV-alginate-PCL group and the other three groups were even greater after 2 months of implantation, at which time the BV and BV/TV of the BMSC-MV-alginate-PCL group had increased to 6.82 ± 0.91 mm^3^ and 24.62% ± 3.55%. In contrast, the BV and BV/TV of the other three groups after 2 months were lower BMSC-alginate-PCL: 3.85 ± 0.60 mm^3^ and 13.97% ± 2.15%, MV-alginate-PCL: 0.24 ± 0.05 mm^3^ and 0.90% ± 0.21%, and Alginate-PCL: 0.18 ± 0.04 mm^3^ and 0.66% ± 0.13% (Fig. 5).

**Figure 6:**
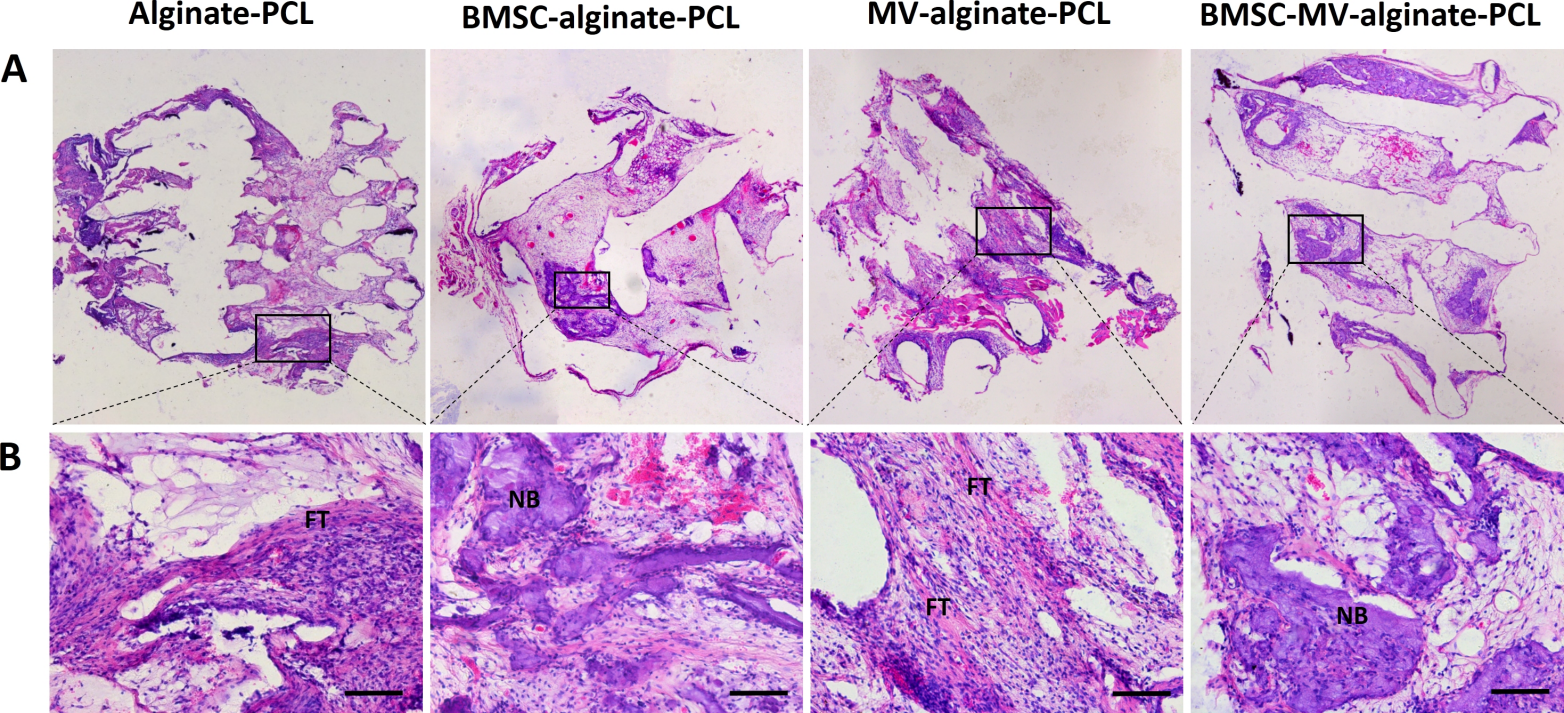
Histological analysis. Representative images of HE staining of the specimens from all of the four groups at low magnification (A) and high magnification (B). Scale bars: 100 µm. NB: new bone; FT: fibrotic tissue.

For further evaluation of the bone formation, HE staining was carried out on samples from each group after 2 months of implantation. As shown in Fig. 6, in the MV-alginate-PCL and Alginate-PCL groups, the constructs were primarily occupied by cord-like fibrotic tissue (high-magnification images in Fig. 6), and very little new bone formation was observed. In the BMSC-MV-alginate-PCL and BMSC-alginate-PCL groups, bone formation with mineralized tissue (high-magnification images in Fig. 6) was observed. Overall, more bone formation was observed in the BMSC-MV-alginate-PCL group than in the other groups.

### MSC-MVs promote vascularization *in vivo*

**Figure 7:**
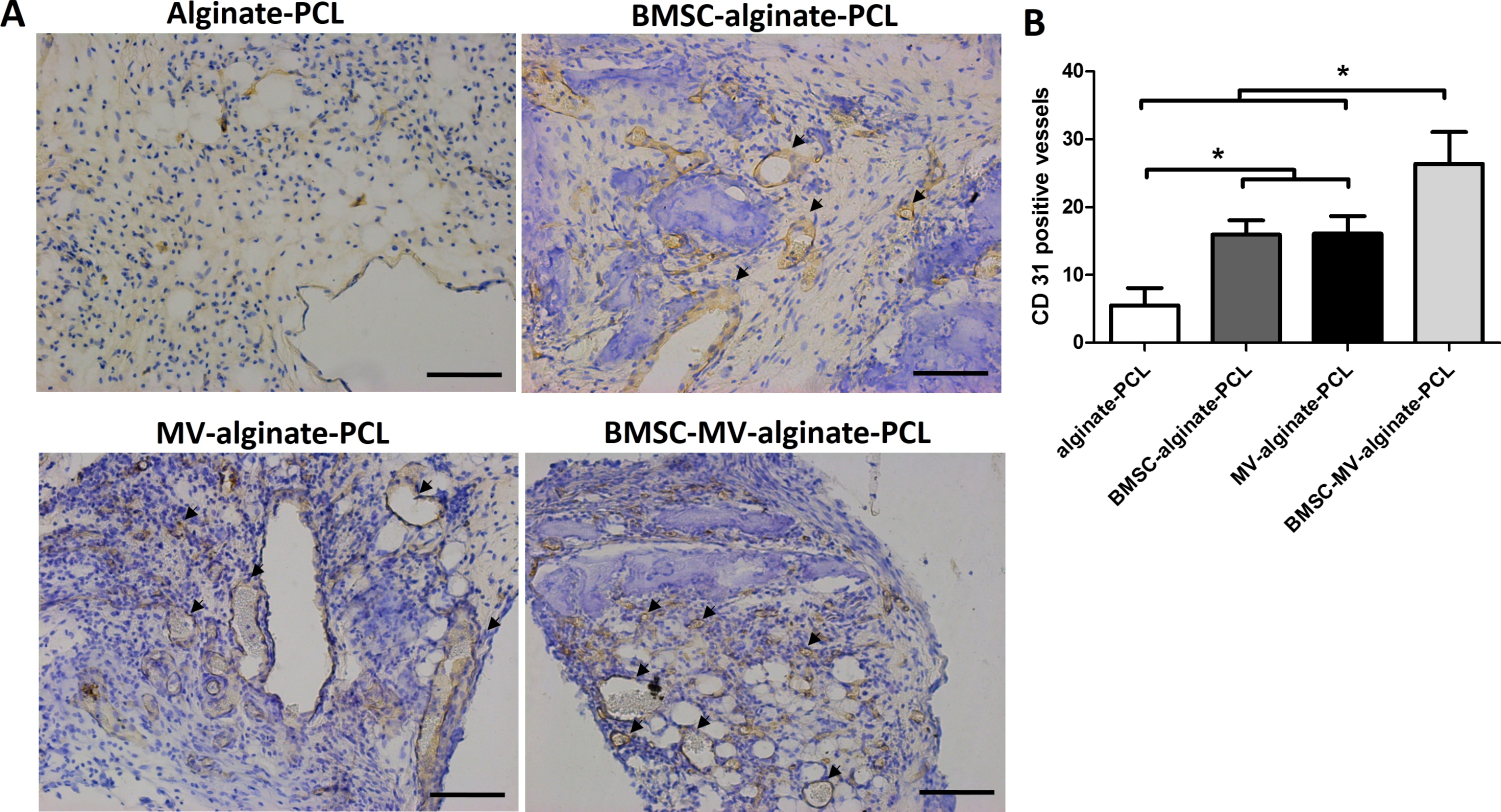
Immunohistochemical analysis. (A) Representative images of CD31 expression in the specimens from each group. Scale bars: 100 µm. (B) Quantitative analysis of CD31-positive vessels in each group after 2 months of implantation. Three samples in each group and five randomly selected views from each sample were analyzed. *p < 0.05.

The ability of MSC-MVs to promote in vivo vascularization was evaluated by quantification of immunohistochemically-stained vessels. Specimens from all four groups showed positive staining for CD31. The mean number of CD31-positive vessels was significantly higher in the BMSC-MV-alginate-PCL group than in the BMSC-alginate-PCL, MV-alginate-PCL, and Alginate-PCL groups (Figs. 7A and 7B). Importantly, in the MV-alginate-PCL group, there was a three-fold increase in the number of CD31-positive vessels compared with that in the Alginate-PCL group (Fig. 7B). These results demonstrated the ability of MSC-MVs to promote vascularization *in vivo*.

## Discussion

The survival rate and repair efficacy of tissue-engineered grafts after implantation in vivo have been shown to be strongly associated with the extent of neovascularization (Saran, Gemini Piperni & Chatterjee, 2014). The aim of this study was to investigate the possibility of incorporating MSC-MVs into an alginate-PCL construct previously developed for bone regeneration, to enhance its therapeutic potential by promoting angiogenesis. Notably, the MV-alginate-PCL construct mixed with osteodifferentiated BMSCs facilitated the most bone formation in the subcutaneous bone formation model in nude mice (Fig. 5). Immunohistochemical staining confirmed the presence of enhanced formation of blood vessels in the MV-alginate-PCL and BMSC-MV-alginate-PCL groups (Fig. 7). These results indicated that it is feasible to incorporate MSC-MVs into alginate-PCL constructs to enhance tissue-engineered bone regeneration by promoting vascularization.

It is widely accepted that synthetic polymer scaffolds are poor at mimicking the natural microenvironment of autologous tissues, especially bone. Appropriate mechanical properties and porosity for the in-growth of vessels, as well as biocompatibility, are required for bone tissue engineering scaffolds (Rath et al., 2012). Alginate-PCL constructs can partly fulfill these requirements. PCL is an FDA-approved bioresorbable polymer for implantation. Porous PCL scaffolds produced by 3D printing with a controlled diameter, range of shape design options, and high mechanical strength have been investigated for their potential in the repair of bone defects (Bao et al., 2015). In addition, alginate, as a natural hydrogel with good biocompatibility and biodegradability, is structurally similar to extracellular matrix and can encapsulate various growth factors (Lee et al., 2013; Rufaihah & Seliktar, 2015). Alginate-PCL constructs have been used as dental and orthopedic implants in several studies (Jang et al., 2013; Kim, Jung & Kim, 2013; Kim & Kim, 2014). However, both PCL and alginate are poor at promoting angiogenesis, which is essential for bone repair.

To the best of our knowledge, the present work describes for the first time the encapsulation of MSC-MVs in an alginate-PCL construct, thus enhancing tissue-engineered bone formation by improving angiogenesis. Several studies have shown that MSC-MVs can mimic the beneficial effects of MSCs, such as the ability to promote angiogenesis (Zhang et al., 2012; Bian et al., 2014; Lopatina et al., 2014). Owing to the fact that MSCs can be incorporated directly into a hydrogel, we hypothesized that MSC-MVs with a phospholipid bilayer may also work in a hydrogel system. Immunohistochemical analyses showed that MSC-MVs improved vascularization in vivo and thereby confirmed our hypothesis. We couldn’t rule out the possibility that MSC-MVs might also promote osteogenic differentiation of the seed cells, so we cultured MSCs in osteogenic differentiation medium in the presence of MSC-MVs or PBS (control) and replaced the medium and MSC-MVs every 3 days for 2 weeks. Then quantitative real-time polymerase chain reaction (qRT-PCR) was performed to detect mRNA expression of osteogenesis-associated genes like Runt-related transcription factor 2 (RUNX2), osteocalcin (OCN), and osteopontin (OPN). The results showed no significant difference in the expression of the osteogenesis-associated genes between the two groups (data not shown), suggesting that the significantly increased newly regenerated bone in the BMSC-MV-alginate-PCL group might be mainly due to the proangiogenic ability of MSC-MVs.

To promote vascularization, various approaches were developed in previous studies (Moon & West, 2008). Delivery of proangiogenic growth factors like VEGF through their encapsulation or incorporation into scaffolds for controlled release was one of the most commonly used approaches. For example, alginate microparticles loaded with VEGF were incorporated into freeze-dried, collagen-based scaffolds to ensure sustained release of bioactive VEGF (Quinlan et al., 2015). However, current approaches for delivering growth factors are often associated with limited success, on account of their uncontrolled release of proteins, short half-life, high cost, and potential safety risks for clinical application (Hettiaratchi et al., 2014). The arteriovenous (A-V) shunt loop strategy, involving a microsurgical approach to achieve an anastomosis between arteries and veins, has been proven to be advantageous for axial vascularization of a scaffold (Rath et al., 2012). Nevertheless, this microsurgical method is also limited by the diameter and distribution of vessels.

Compared with the above-mentioned approaches, MSC-MVs have several inherent advantages. First, they are enriched in angiogenesis-promoting biomolecules and angiogenesis-related mRNAs and miRNAs (Chen et al., 2014; Eirin et al., 2014). Specifically, Chen et al. (2014) reported that MVs released by human umbilical cord-derived MSCs contained a variety of angiogenesis-promoting factors, including VEGF, interleukin-6, basic fibroblast growth factor, angiogenin, and monocyte chemotactic protein-1. In addition, Eirin et al. (2014) discovered that MVs released by porcine adipose tissue-derived MSCs preferentially expressed mRNAs and miRNAs involved in angiogenesis, which might induce genetic alteration of the recipient cells. Second, the isolation of MSC-MVs is more economical than the use of expensive growth factors like VEGF. MSCs are usually greatly expanded because large numbers of seed cells are essential to construct a tissue-engineered graft. Since MSCs secrete large numbers of MVs during culture, recycling of their culture supernatant, which is usually discarded during cell passaging, for the isolation of MSC-MVs would avoid unnecessary waste and reduce the cost. Third, accumulating evidence proves that allogeneic and even xenogeneic MVs have little or no toxicity and immunogenicity in immune-competent animals (Gyorgy et al., 2015). Furthermore, it has been suggested that MV-induced cell-to-cell communication can occur across species (Gatti et al., 2011; Aliotta et al., 2012), which might solve the problem of the severe shortage of appropriate donors.

Although MSC-MVs hold great potential in bone tissue engineering applications, there are still many issues needed to be addressed. Firstly, the exact mechanism underlying the proangiogenic effect of MSC-MVs remains unclear. Future studies could investigate the genetic and epigenetic changes in target cells induced by MSC-MVs and the bioactive cargos enclosed in MSC-MVs. Secondly, a number of studies have demonstrated that MSC-MVs could exert anti-apoptotic effects on injured cells (Bruno et al., 2012; Lin et al., 2014). Since the seed cells in the core area of the scaffold often suffer from hypoxia and poor nutrient supply due to the lack of vascularization (Moon & West, 2008), it is worthwhile to investigate whether MSC-MVs may exert other beneficial effects on the seed cells in future. Thirdly, we did not compare the effects of MSC-MVs with proangiogenic growth factors in the present study, future study could investigate whether MVs could replace growth factors to achieve better outcomes. Although many issues remain to be addressed, the MV-alginate-PCL construct described herein represents a promising approach for promoting vascularization in tissue-engineered grafts. This composite graft has enormous potential for tissue engineering and regenerative medicine, and may be applied to the regeneration of other tissues and organs.

## Conclusions

The present study developed a novel MV-alginate-PCL construct that takes advantage of the proangiogenic properties of MSC-MVs. MVs could be harvested from the culture medium of MSCs during their expansion and incorporated into alginate-PCL constructs. When combined with osteodifferentiated MSCs, theses constructs led to notable increases in vessel formation and tissue-engineered bone regeneration in a subcutaneous bone formation model in nude mice. Taking all of the findings into consideration, these constructs may offer a novel proangiogenic strategy for bone tissue engineering applications.

## Supplemental Information

10.7717/peerj.2040/supp-1 Figure S1 Characterization of BMSC-MV-alginate-PCL constructs. Representative images of the core area (A) and margin area (B) of the BMSC-MV-alginate-PCL constructs observed by phase-contrast microscopy, scale bars: 200 µm. Representative images of the BMSC-MV-alginate-PCL constructs observed by confocal microscopy (C and D), scale bars: 100 µm.

## Additional Information and Declarations

### Data Availability

The following information was supplied regarding data availability: Figshare: https://figshare.com/s/cff0b8d436f09c71591d.
