# Supplementary figures and images for: Development of an angiogenesis-promoting microvesicle-alginate-polycaprolactone composite graft for bone tissue engineering applications
Source: PeerJ. 2016 May 19;4:e2040. doi: 10.7717/peerj.2040 (PMC4878377; doi:10.7717/peerj.2040)

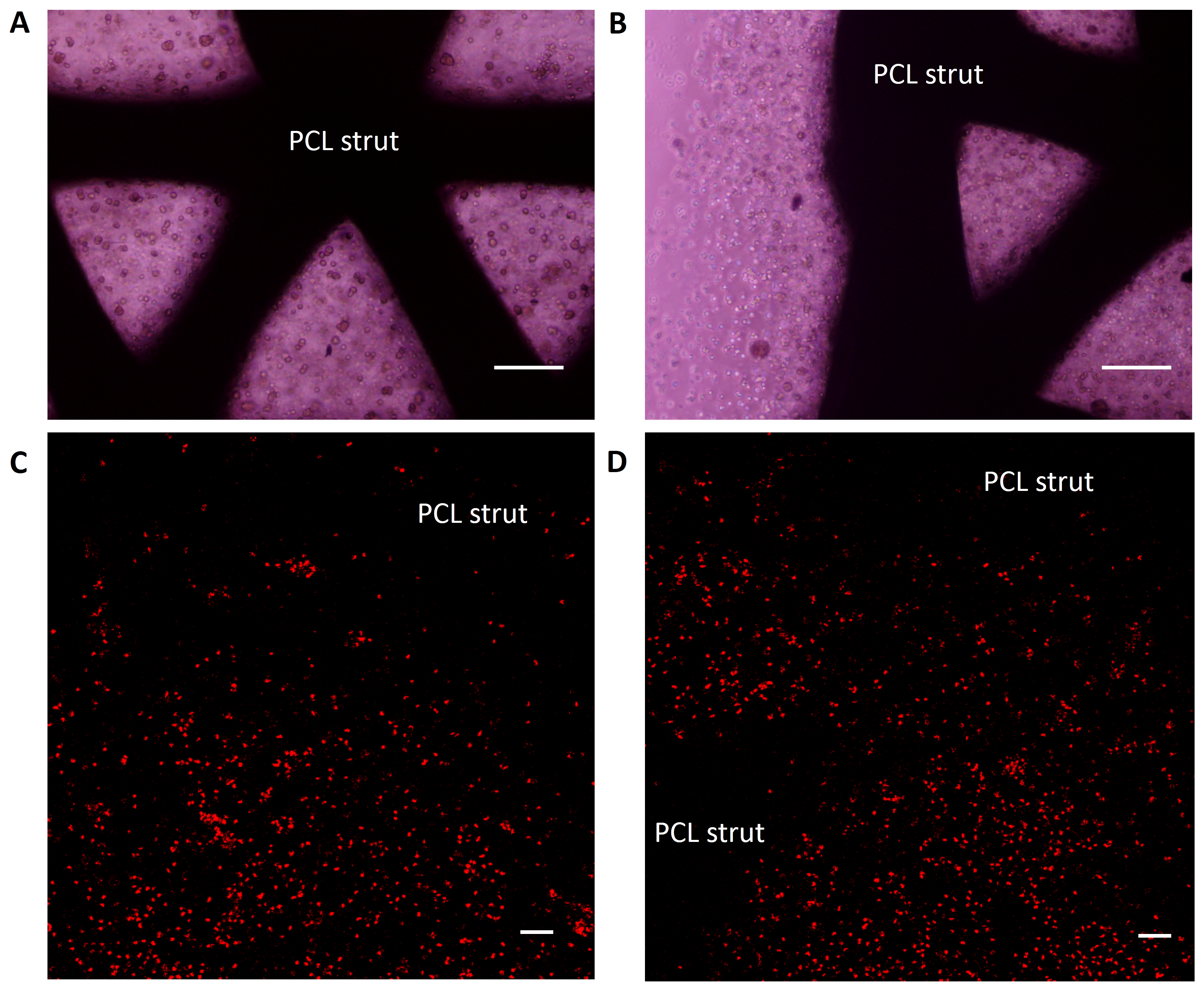

Supplement: Figure S1 — Characterization of BMSC-MV-alginate-PCL constructs. Representative images of the core area (A) and margin area (B) of the BMSC-MV-alginate-PCL constructs observed by phase-contrast microscopy, scale bars: 200 µm. Representative images of the BMSC-MV-alginate-PCL constructs observed by confocal microscopy (C and D), scale bars: 100 µm. [file peerj-04-2040-s001.png]
